# Strain-Engineered Phase Diagrams in (SrTiO_3_)_8_/(BaTiO_3_)_8_ Superlattices: Toward Néel Skyrmions and Energy Storage

**DOI:** 10.3390/nano16100582

**Published:** 2026-05-10

**Authors:** Tangrui Dan, Wenhua Zhang, Fengjuan Yang, Jiong Wang, Yingxin He, Pingping Wu

**Affiliations:** 1 Department of Materials Science and Engineering, Xiamen Institute of Technology, Xiamen 361021, China; 2 The Higher Educational Key Laboratory for Flexible Manufacturing Equipment Integration of Fujian Province, Xiamen Institute of Technology, Xiamen 361021, China; funfunyang@126.com; 3 State Key Laboratory of Powder Metallurgy, Central South University, Changsha 410083, China; wangjionga@csu.edu.cn; 4 Key Laboratory of Information Functional Material for Fujian Higher Education, Quanzhou Normal University, Donghai District, Quanzhou 362000, China; 5 Department of Physics, Quanzhou Normal University, Donghai District, Quanzhou 362000, China

**Keywords:** substrate, domain structure, polar skyrmion, superlattices, phase diagram, phase field model

## Abstract

Misfit strain between the substrate and the superlattice plays a critical role in determining the domain configurations and ferroelectric properties of superlattice heterostructures. This implies that a proper substrate material can be selected to design and tailor the domain structure and the properties of the superlattice. However, the influence of the substrate-induced strain on domain structures and polarization switching processes under different substrates is difficult to observe experimentally. In this study, we employ the phase-field method to explore the effects of different substrates on the domain patterns and switching properties of superlattice heterostructures. A phase diagram mapping substrate lattice parameters against the electric field was constructed for ferroelectric (SrTiO_3_)_8_/(BaTiO_3_)_8_ superlattice thin films. Néel-type skyrmion structures were observed under an applied electric field and could be stabilized upon field removal. Two distinct switching modes were also identified, depending on the substrate. Additionally, we observed that misfit strain-induced hysteresis linearization enhances both recoverable energy density and energy storage efficiency, suggesting that superlattice heterostructures hold promise for energy storage applications. Our findings provide new insights into the switching mechanisms of superlattice structures and pave the way for designing next-generation functional nanoelectronic devices.

## 1. Introduction

The ferroelectric skyrmions in superlattice structures have garnered great attention due to their potential applications in next-generation nanoelectronic devices, particularly for high-density and high-speed data storage devices [1,2,3,4,5,6,7,8,9,10,11,12,13]. These skyrmions exhibit many exotic physical phenomena, like negative permittivity [5], chirality [6], and improper ferroelectricity [7]. The formation of skyrmion structures and their associated ferroelectric/dielectric properties in superlattice heterostructures are governed by various factors, including temperature, applied electric fields, stacking sequences, thin film thickness, and the substrate [12]. This dependence enables the tailoring and design of ferroelectric superlattice properties through tuning these factors. These extensive studies highlight the broad design space available for exploring behaviors beyond novel phases and for enhancing ferroelectric/dielectric properties in oxide superlattices.

Current research mainly focuses on PbTiO_3_ (PT)-based materials [2,3,4,5,6,7,12,13], which can be attributed to their tetragonal structure and high spontaneous polarization at room temperature, promoting the formation of stable skyrmion structures. However, due to the environmental concerns associated with lead-based PT-based superlattices, widely investigated BT-based superlattices have emerged as excellent lead-free alternatives. Recent studies have reported skyrmion structures within BaTiO_3_/SrTiO_3_ (BT/ST) superlattices, BaTiO_3_–SrTiO_3_ composites and rhombohedral BaTiO_3_ [14,15,16,17].

As a superlattice, (BaTiO_3_)_n_/(SrTiO_3_)_n_ has been widely investigated and used for its exotic domain structures, relaxor-like behavior, and highly tunable ferroelectric properties [18,19,20,21,22,23,24,25,26,27,28]. Selecting a proper substrate is a key step in the experimental growth of superlattice heterostructures. Domain engineering via substrates is a powerful approach for tailoring functional ferroelectric materials. For example, Ishibe et al. intentionally controlled domain size and defect density using substrates with low symmetry [29]. Chen et al. introduced a dielectric layer to control the domain patterns of BiFeO_3_ [30]. Winchester et al. used miscut substrates to effectively generate striped or mosaic domain patterns [31]. Additionally, specific studies focus on the control of polar topological patterns [32] and domain orientations [33]. For ST/BT superlattice structures, the interfacial coherency between the superlattice and the substrate has a strong influence on the domain structure, hysteresis loops, and other ferroelectric properties [18,19,20].

Early studies of ST/BT superlattices grown on substrates focused on their basic physical properties, including Curie temperature, phase structures, ferroelectric domain patterns, lattice constants, dynamic properties, and strain relaxation [18,19,20,21,22]. Recently, the polarization evolution and topological structures of ST/BT superlattices have attracted significant interest due to their potential applications in memory devices. Experimentally, Bein et al. utilized rapid in situ synchrotron X-ray diffraction to monitor the evolution of ferroelectric domains during the growth of ST/BT superlattices [23]. Furthermore, rotational polarization nanotopological structures [24], three-dimensional polarization vortex structures [25], and skyrmion-like structures [14] have been observed in ST/BT superlattices. These works demonstrate the feasibility of controlling the different degrees of freedom associated with these topologies, which could lead to technological applications similar to (or distinct from) those of magnetic skyrmions. Studies on switching and dielectric properties have revealed that ST/BT superlattices exhibit relaxor-like behavior [26]. Very recently, negative capacitance properties have been reported in ST/BT superlattice structures, implying that these superlattices hold potential for low-power microelectronics applications [27,28].

The phase-field method has proven to be a powerful tool for predicting the domain structure, phase diagram, and domain evolution process of ferroelectric skyrmions [3,4,5,8,12,13,14,34,35,36,37,38]. In our previous work [14], we predicted domain structures under fully relaxed strain conditions and discovered skyrmion structures when the electric field is removed. However, the effects of the substrate on the domain patterns of superlattice heterostructures are rarely studied, and the intrinsic mechanism of the polarization switching process under substrate effects remains unclear for the design of superlattice systems.

In this work, we investigated the influence of substrates on the domain structures and field-driven evolution of ST_8_/BT_8_ superlattices at room temperature using phase field simulations. The superlattice was assumed to be grown on six substrates: SrTiO_3_, DyScO_3_, TbScO_3_, GdScO_3_, SmScO_3_, and BaTiO_3_. Several polarization topological configurations are observed in the superlattice structure. By constructing a substrate lattice constant-electric field phase diagram, we established the conditions for obtaining stable ferroelectric skyrmions. Furthermore, we visualized the polarization switching mechanisms in superlattices grown on different substrates under external fields. Notably, we present the unipolar P-E loops for the superlattice structures on different substrates. As the substrate lattice parameter increases, the hysteresis loop exhibits a linear characteristic, thereby identifying the ST_8_/BT_8_ superlattices on specific substrates as promising candidates for energy storage applications.

## 2. Simulation Method

The lattice phase field model is used to simulate a ST_8_/BT_8_ ferroelectric superlattice structure [39], and order parameter *η* is chosen to distinguish between the BT and ST phases. The value of *η*(*r*) is set to be

(1) $$\eta (r)=\left\{\begin{array}{c} 0,\;\;\;\;\;\;\;\;ST\;phase\\ 0.5,\;\;\;\;\;interface\\ 1,\;\;\;\;\;\;\;\;\;BT\;phase \end{array}\right.$$

For simplicity, *η* is considered static in this simulation.

To describe the domain structure of a ferroelectric superlattice, the spontaneous polarization *P_i_* (*r*, *t*) (*i* = 1, 2, and 3) is chosen to be another order parameter. Therefore, the spatial evolution of the ferroelectric polarization distribution is governed by the time-dependent Ginzburg–Landau equation:

(2) $$\frac{\partial P_{i}(r,t)}{\partial t}=-L\frac{\delta F}{\delta P_{i}(r,t)},$$

where *t* is time, *L* is the dynamic coefficient, and *F* is the free energy of the superlattice system. According to the Landau–Ginzburg–Devonshire theory, the free energy *F* is expressed as

(3) $$F=\int_{V}{(f}_{Land}+f_{grad}+f_{elec}+f_{elas})\;dV,$$

where *f_Land_*, *f_grad_*, *f_elec_*, and *f_elas_* represent the Landau potential, gradient, electrostatic, and elastic energy densities, respectively; *V* is the total volume.

The Landau potential energy density can be expressed as

(4) $$f_{Land}=\alpha_{i}\left(r\right)P_{i}^{2}+{\alpha_{ij}\left(r\right)P_{i}^{2}P}_{j}^{2}+{\alpha_{ijk}\left(r\right)P_{i}^{2}P_{j}^{2}P}_{k}^{2}+{\alpha_{ijkl}\left(r\right)P_{i}^{2}P_{j}^{2}P_{k}^{2}P}_{l}^{2},$$

where *α_i_*(*r*), *α_ij_*(*r*), *α_ijk_*(*r*), and *α_ijkl_*(*r*) are the Landau expansion coefficients. Note that here Einstein’s summation rule and Voigt’s matrix notation are employed for the tensors. The value of the Landau coefficients is correlated with the order parameter *η*(*r*) and can be written as *α*(*r*) = *α*_BT_**·***η*(*r*) + *α*_ST_**·**(1 − *η*(*r*)), where *α*_BT_ and *α*_ST_ represent the Landau coefficients of BaTiO_3_ and SrTiO_3_ phase in the superlattice.

The gradient energy density is related to the formation of ferroelectric domain wall and is given by

(5) $$f_{grad}=\frac{1}{2}G_{ijkl}\frac{\partial P_{i}}{\partial x_{j}}\frac{\partial P_{k}}{\partial x_{l}},$$

where *G_ijkl_* is the gradient energy coefficient. The electrostatic energy density can be written as

(6) $$f_{elec}=-\frac{1}{2}\varepsilon_{b}\varepsilon_{0}E_{i}^{2}-E_{i}P_{i},$$

where ε_b_ is the background dielectric constant, and *E_i_* is the external electric field.

The elastic energy density is calculated based on Khachaturyan’s micro-elastic theory [40]

(7) $$f_{elas}=\frac{1}{2}C_{ijkl}\left(\varepsilon_{ij}-Q_{ijkl}P_{k}P_{l}\right)\left(\varepsilon_{kl}-Q_{klij}P_{i}P_{j}\right),$$

where *C_ijkl_* and *Q_ijkl_* are elastic stiffness and electrostrictive coefficient tensors, and *ε_ij_* is the total strain, which can be decomposed into two parts: homogeneous strain and heterogenous strain, i.e.,

(8) $$\varepsilon_{ij}=\bar{\varepsilon_{\mathrm{ij}}}+\delta \varepsilon_{ij}\left(r\right),$$

where $\bar{\varepsilon_{\mathrm{ij}}}$ is the homogenous strain, a uniform macroscopic strain that determines the macroscopic shape deformation of the materials. For the superlattice structure, the macroscopic in-plane mismatch strain is the homogeneous strain, which is determined by the lattice parameter mismatch between the substrate material and the superlattice material

(9) $$\bar{\varepsilon_{11}}=\bar{\varepsilon_{22}}=\frac{a_{sub}-a_{sup}}{a_{sup}},$$

where *a*_sub_ and *a*_sup_ represent the lattice parameter of the substrate and superlattice material. For ST/BT superlattice structure, a_sup_(r) = *a*_BT_**·***η*(r) + *a*_ST_**·**(1 − *η*(r)), and *a*_BT_ and *a*_ST_ represent the lattice constant of BaTiO_3_ and SrTiO_3_, respectively. Six different substrates are employed in this work, including SrTiO_3_, DyScO_3_, TbScO_3_, GdScO_3_, SmScO_3_, and BaTiO_3_. The lattice parameters of these materials can be found in Refs. [41,42]. Table 1 lists the lattice parameters used in this simulation and the calculated mismatch strain in the ST and BT layers of the superlattice.

Noticing that the homogeneous strain along the out-of-plane direction is relaxed, the homogenous strain should therefore be calculated as

(10) $$\bar{\varepsilon_{33}}=\int_{V}Q_{ijkl}P_{k}P_{l}d^{3}x$$

The heterogeneous strain part is related to the local displacement *u*(r) and can be calculated using the usual elasticity relation:

(11) $$\delta \varepsilon_{ij}\left(r\right)=\frac{1}{2}\left(\frac{\partial u_{i}}{\partial r_{j}}+\frac{\partial u_{j}}{\partial r_{i}}\right)$$

Substituting Equations (9)–(11) into Equation (8) can calculate the total strain of the superlattice system. Hence, the elastic energy can be obtained using Equation (7). Subsequently, the total free energy of the superlattice system is calculated using Equations (3)–(7). Finally, the domain structure and polarization distribution of the ferroelectric superlattice are obtained by solving Equation (2) via the semi-implicit Fourier spectral method [43]. The Landau energy coefficients, gradient energy coefficients, elastic energy coefficients and electrostrictive coefficients are listed in Table 2, consistent with our previous studies [14,44]. The periodic boundary conditions are employed along all the *x*, *y* and *z* directions.

All simulations were performed at 300 K (room temperature). The thermal expansion effect is not taken into consideration in this work, since the thermal expansion rates of these oxides do not differ significantly in the 200–400 K range, and the mismatch strain between the substrate and the superlattice undergoes only minor changes [45]. The ST_8_/BT_8_ superlattice is modeled as fully commensurate with the substrate and is assumed to be defect-free in this simulation, as the thickness of the BaTiO_3_ layer is approximately 3.2 nm, which is well below the critical thickness observed in experiments [46,47].

## 3. Results and Discussion

**Figure 1:**
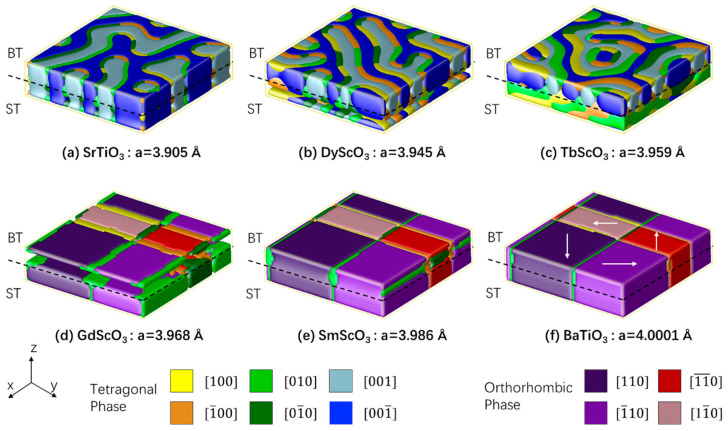
Simulated ferroelectric domain structures of superlattice ST_8_/BT_8_ on various substrates: (**a**) SrTiO_3_, (**b**) DyScO_3_, (**c**) TbScO_3_, (**d**) GdScO_3_, (**e**) SmScO_3_, and (**f**) BaTiO_3_. The lattice parameters are indicated in the figure.

Figure 1a–f illustrates the simulated domain structures of the superlattice ST_8_/BT_8_ on six different substrates: SrTiO_3_, DyScO_3_, TbScO_3_, GdScO_3_, SmScO_3_, and BaTiO_3_, respectively. The simulation cell is discretized with 64Δ*x* × 64Δ*y* × 16Δ*z* grids, where Δ*x* = Δ*y* = *a*_sub_, Δ*z*(r) = *a*_BT_·*η*(r) + *a*_ST_·(1 − *η*(r)). At *E*_z_ = 0, the BT_8_/ST_8_ superlattice structure grown on SrTiO_3_ exhibits a labyrinth domain pattern, as shown in Figure 1a. The BT phase was constrained by the compressive stress induced by the SrTiO_3_ substrate and exhibited tetragonal c+/c− domains. In the absence of misfit strain, polarization in the ST phase was induced by the electrostatic effect. With increasing substrate lattice parameter, the domain wall width of the labyrinth structure expands, particularly at the interface between the BT and ST phases. On the TbScO_3_ substrate, the BT phase retains a tetragonal labyrinth structure, while the polarization in the ST phase begins to rotate toward the in-plane direction. On the GdScO_3_ substrate, which has a larger lattice parameter, the ST phase of the superlattice first transforms into an orthorhombic phase, accompanied by a significant decrease in the BT phase polarization. For substrates with even larger lattice parameters, such as SmScO_3_ and BaTiO_3_, an orthorhombic phase is also observed in the BT phase to minimize the depolarization field.

**Figure 2:**
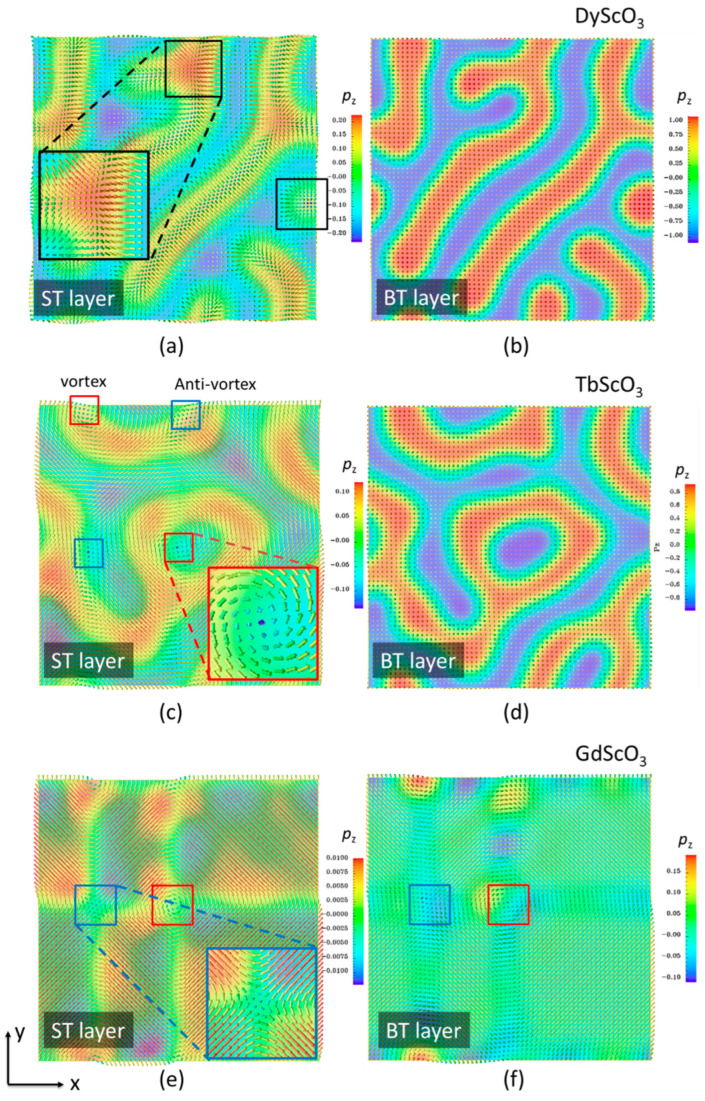
Vector plot of the ST layer (**left column**) and the BT layer (**right column**) in ST_8_/BT_8_ grown on substrates of DyScO_3_ (**a**,**b**), TbScO_3_ (**c**,**d**), and GdScO_3_ (**e**,**f**). The black box shows the rosette structures. The red box and blue box show typical vortex and antivortex structures, respectively.

Several topological structures were observed in the superlattice structures. Figure 2a–f illustrates the vector plots at the center of the BT and ST layers of the superlattice grown on DyScO_3_, TbScO_3_, and GdScO_3_ substrates, respectively. For the superlattice grown on DyScO_3_, the polarization in the BT layer remains oriented along the +/−z direction. However, in the ST layer, the polarization lies in-plane owing to the tensile in-plane strain, exhibiting rosette-like structures, as highlighted in the black boxes (Figure 2a). In the ST layer of the superlattice grown on TbScO_3_, vortex (red box) and anti-vortex (blue box) pairs were observed (Figure 2c), implying that a phase transformation from the tetragonal phase to the orthorhombic phase occurs. For the superlattice grown on the GdScO_3_ substrate, vortex-antivortex pairs were simultaneously observed in both the ST and BT layers (Figure 2e,f), indicating that the entire structure has transformed to an orthorhombic phase.

**Figure 3:**
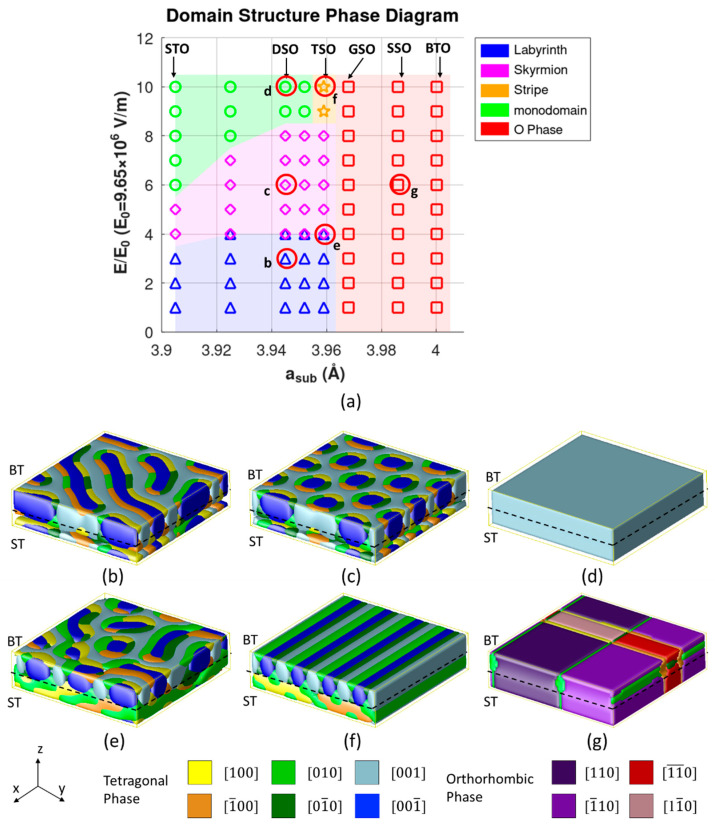
(**a**) Phase field-predicted domain structure phase diagram showing six types of structures in red circles. (**b**–**g**) show labyrinth, skyrmion, stripe, monodomain, mixed labyrinth and skyrmion, and orthorhombic phase structures, respectively.

To achieve stable ferroelectric topological structures, the whole structure was initially polarized under an electric field of 10*E*_0_(~9.65 × 10^7^ V/m), then we gradually decreased the external field to zero and examined the resulting domain structures. Distinct domain structures were observed in the simulation. Figure 3a shows a phase diagram of the polar textures constructed by the phase field model. The horizontal axis of the phase diagram represents the substrate lattice constant *a*, while the vertical axis denotes the maximum applied electric field *E*_z_. The six typical simulated domain patterns are illustrated in Figure 3b–g, including labyrinth, skyrmion, stripe, monodomain, mixed labyrinth-skyrmion, and orthorhombic structures. To better understand the formation mechanism of these polarization topological structures, the evolution of the domain structure and the polarization distribution was further examined. Based on the phase diagram, two distinct modes of ferroelectric domain structure evolution under an electric field were observed on different substrates. In Mode I, the domain structure evolution undergoes three distinct stages with increasing electric field: labyrinth, skyrmion, and monodomain. According to the phase diagram, when the substrate lattice constant is less than 3.9658 Å (as in SrTiO_3_, DyScO_3_, and TbScO_3_ substrates), the domain structure evolution consistently follows the characteristics of Mode I.

**Figure 4:**
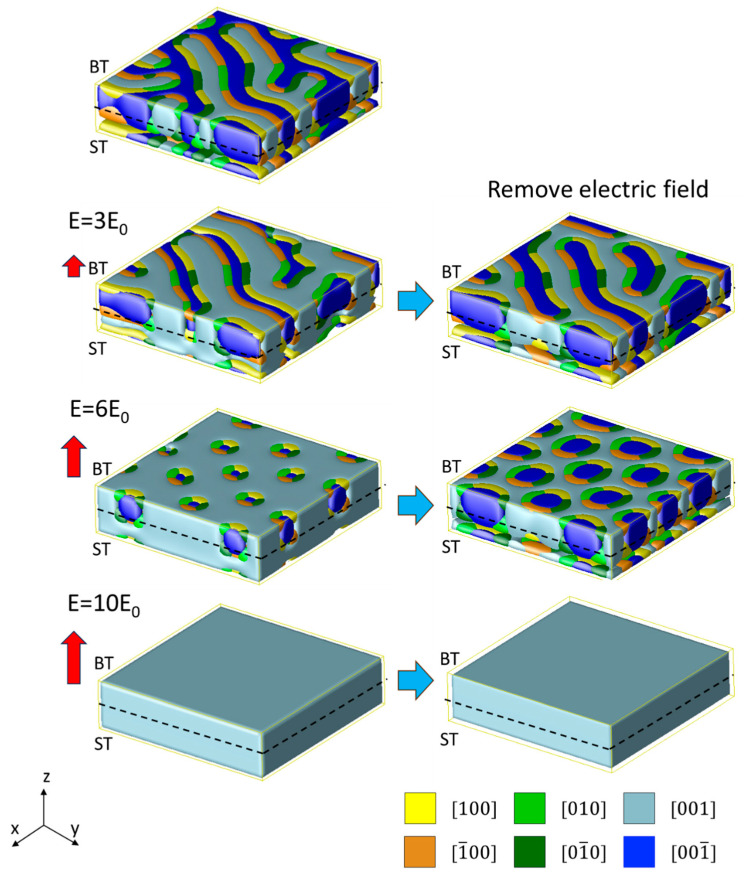
Domain evolution process of the ST_8_/BT_8_ superlattice grown on DyScO_3_ under an electric field applied along the +z direction (Mode I). Labyrinth, skyrmion, and monodomain structures are obtained after applying and subsequently removing electric fields of 3*E*_0_, 6*E*_0_ and 10*E*_0_, respectively.

Figure 4 illustrates the typical domain structure evolution for Mode I, where the superlattice is grown on a DyScO_3_ substrate. The initial domain structure exhibits a typical labyrinth domain pattern; similar structures have been reported experimentally in PT/ST superlattices [3,4,13] and ultrathin PbTiO_3_ films [48,49]. As *E*_z_ increases, the −c domains begin to shrink while the +c domains expand, and the labyrinth pattern can be maintained. Upon removing the external field at this stage, the labyrinth pattern is preserved but exhibits slight variations compared to the initial structure. Further increasing *E*_z_ to 6*E*_0_, a bubble-like skyrmion structure emerges, exhibiting a well-ordered hexagonal close-packed pattern like our previously reported results [14]. When the external field is removed, these bubble-like skyrmion domains expand while maintaining their original structures because of topological protection. Finally, when the external field reaches 9*E*_0_, the skyrmion structures are destroyed, and the whole superlattice system transforms into a single +c domain structure. At this stage, removing the external field leaves the monodomain structure unchanged, while the *P*_z_ component decreases, exhibiting hysteresis behavior relative to the external field.

**Figure 5:**
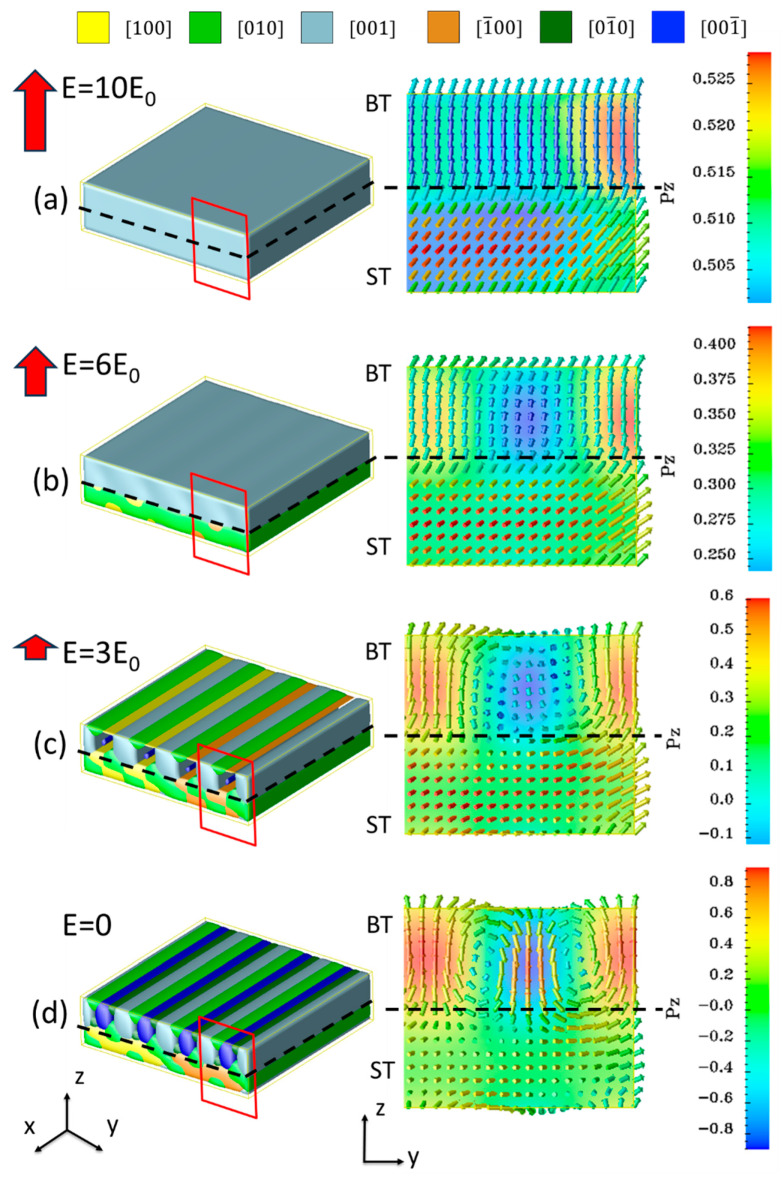
The stripe domain generation process in the ST_8_/BT_8_ grown on the TbScO_3_ substrate. (**a**–**d**) represent the domain structures (**left**) and corresponding polarization vector plots (**right**) during the electric field decreasing process at *E*_z_ = 10*E*_0_, 6*E*_0_, 3*E*_0_ and 0.

It is worth noting that a stripe domain structure was observed for the ST_8_/BT_8_ superlattice grown on the TbScO_3_ substrate. Increasing the field to *E*_z_ ≥ 9*E*_0_ results in a monodomain structure; however, a stripe domain structure formed spontaneously when the external electric field was removed. Figure 5a–d illustrates the domain structure evolution and the polarization vector distribution during this process. First, at the electric field *E*_z_ = 10*E*_0_, the polarization in the BT phase aligns fully along the +z axis, while that in the ST phase maintains an in-plane component (Figure 5a). Further reducing the external field, the depolarization field leads to polarization inhomogeneity in the BT phase (Figure 5b). On the other hand, the introduction of new domain walls can reduce the overall electrostatic energy of the system. The competition between the energies promotes the nucleation of −c domains in the BT phase (Figure 5c), which subsequently grow as the electric field is further reduced, eventually leading to the emergence of a stripe domain structure (Figure 5d).

**Figure 6:**
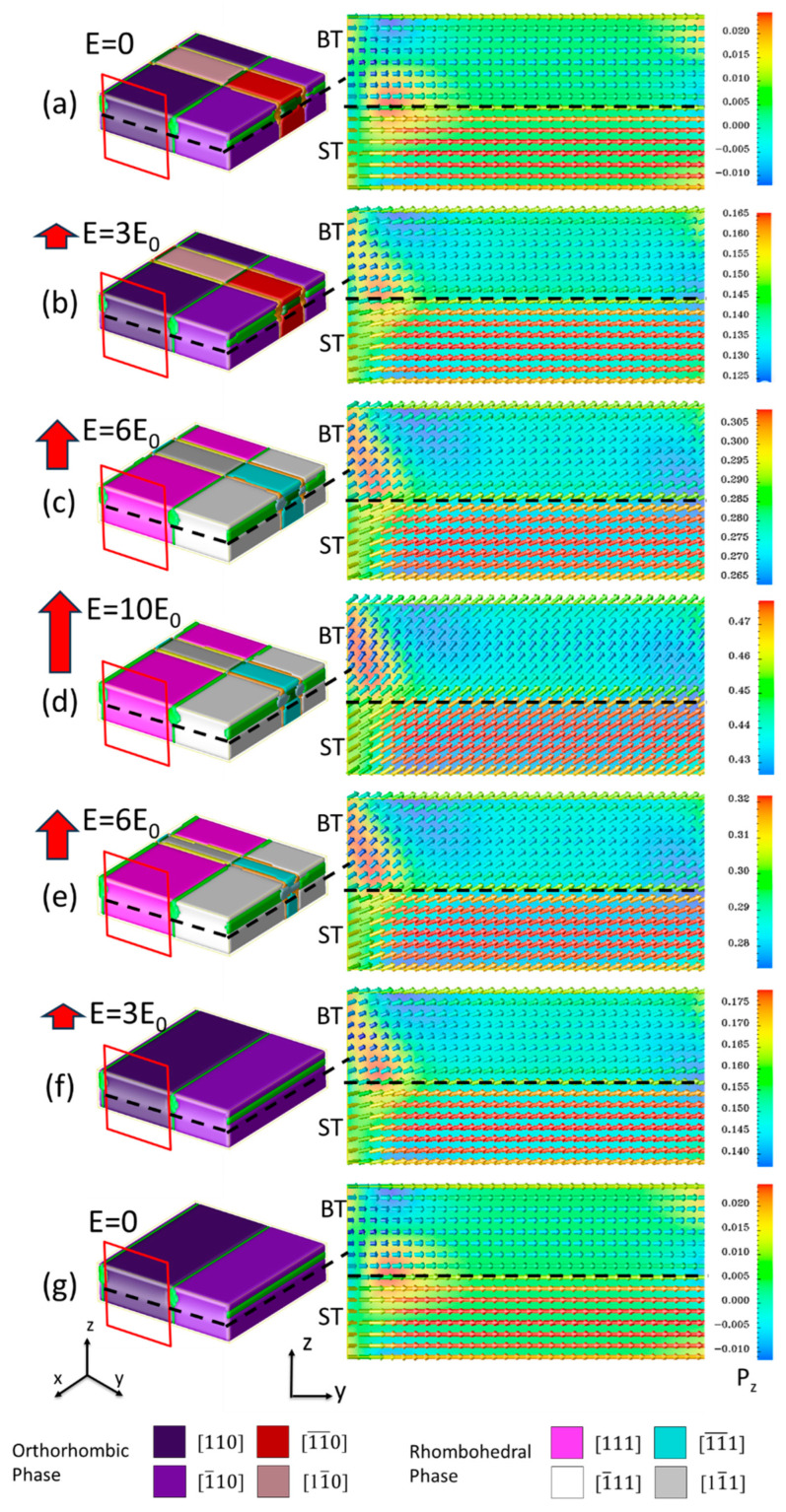
The domain structure evolution of the unipolar process of domain structure (**left column**) and vector plot (**right column**) of the ST_8_/BT_8_ grown on SmScO_3_ (Mode II). (**a**–**d**) show the domain structures and polarization vectors at increasing electric field strengths of 0, 3*E*_0_, 6*E*_0_, and 10*E*_0_, respectively. (**e**–**g**) show the corresponding distributions during the electric field decreasing process at 6*E*_0_, 3*E*_0_, and 0, respectively.

With the further increase of the substrate lattice constant, the elastic energy induced by the substrate drives a phase transition from the tetragonal to the orthorhombic phase in the superlattice. Specifically, our simulations indicate that once the substrate lattice constant is larger than 3.9658 Å, the switching mode of the superlattice structure exhibits a distinctly different mode, which we denote as Mode II. In Mode II, the variation of polarization is primarily caused by the rotation of the polarization vectors. Figure 6 illustrates the domain structure evolution and the corresponding polarization vector plots for the ST_8_/BT_8_ superlattice grown on the SmScO_3_ substrate under an external electric field. At *E*_z_ = 0, the polarization in both the BT and ST phases is oriented in the in-plane direction. As the external field increases, the rise in polarization stems mainly from polarization rotation rather than from the motion of 180° domain walls. When *E*_z_ ≥ 6*E*_0_, an orthorhombic to rhombohedral phase transition is observed as the polarization rotates from the in-plane <110> direction to the +z-oriented <111> direction. Notably, the polarization rotation process is fully reversible; when the external field is removed, the polarization rotates back to its original state, ultimately recovering an in-plane orthorhombic phase similar to the initial configuration.

**Figure 7:**
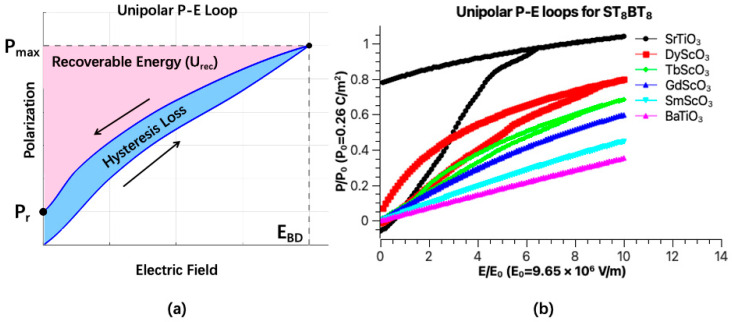
(**a**) A schematic diagram of the hysteresis loop with key parameters for evaluating energy storage performance and (**b**) phase field simulated unipolar P-E loops for ST_8_/BT_8_ superlattice thin films grown on various substrates.

The reversible domain structure evolution depicted in Figure 6 corresponds to a slim hysteresis loop with low hysteresis loss, which suggests that the superlattice structure on some specific substrates has great potential for energy storage applications. Figure 7a shows a schematic figure of the ferroelectric hysteresis loop with corresponding key parameters for evaluating energy storage performance. The energy density of ferroelectric energy storage devices is strongly related to the breakdown electric field *E*_BD_. A higher *E*_BD_ is necessary for device reliability and performance, as it allows the material to withstand stronger external electric fields and directly contributes to a higher recoverable energy density. The recovered energy density *U*_rec_ quantifies the energy storage capability and is calculated as follows:

(12) $$U_{\mathrm{rec}}=\int_{P_{r}}^{P_{max}}EdP,$$

where *P*_max_ and *P*_r_ represent the maximum polarization and the remanent polarization of the hysteresis loop, respectively. In Figure 7a, the pink area above the polarization hysteresis loop represents the recoverable energy density *U*_rec_ and the light blue area inside the loop represents the energy loss *U*_loss_ of the material. Therefore, the energy storage efficiency can be expressed as

(13) $$\eta =\frac{U_{rec}}{U_{rec}+U_{loss}}.$$

The unipolar P-E loops for the ST_8_/BT_8_ superlattice on different substrates are presented in Figure 7b. In this process, an external electric field was applied along the out-of-plane direction to polarize the system, and then gradually reduced to zero. For the superlattice structure grown on SrTiO_3_, a high hysteresis loss was observed in the P-E loop, which can be attributed to the strong compressive elastic strain in the BT layer. Please note that the polarization in the ST layer was totally induced by the depolarization field. As the substrate lattice parameter increases, both the remanent polarization and hysteresis loss dramatically decrease, accompanied by a reduction in *P*_max_. The shape of the P-E loops becomes slim and linear. This linear response is primarily driven by the polarization rotation mechanism in superlattices grown on substrates with larger lattice parameters.

**Figure 8:**
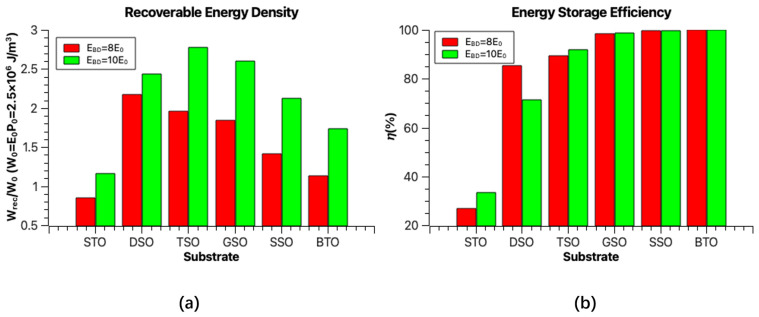
Comparison of the (**a**) recoverable energy and (**b**) energy storage efficiency for ST_8_/BT_8_ superlattice grown on various substrates. The red bar and green bar represent the predicted values assuming *E*_BD_ = 8*E*_0_ and 10*E*_0_, respectively. STO, DSO, TSO, GSO, SSO and BTO refer to the substrate materials SrTiO_3_, DyScO_3_, TbScO_3_, GdScO_3_, SmScO_3_, and BaTiO_3_, respectively.

The recoverable energy density and the energy storage efficiency of the superlattice can be calculated through Equations (12) and (13), as shown in Figure 8a,b, respectively. For the ST_8_/BT_8_ superlattice grown on an SrTiO_3_ substrate, the large hysteresis loss resulting from the hysteresis loop leads to low recoverable energy (*U*_rec_) and energy efficiency (*η*). However, with the increased substrate lattice parameter, the hysteresis loop of the superlattice films becomes slim and shows a linear response with lower hysteresis loss, resulting in a significant enhancement in both the recoverable energy and efficiency.

According to Lupi et al.’s experimental work [26], a maximum electric field of 750 kV cm^−1^ was applied to investigate the switching properties of ST_n_/BT_n_ superlattice structures. In this work, we assume a similar electric breakdown field *E*_BD_ = 8*E*_0_~770 kV cm^−1^. The ST_8_/BT_8_ superlattice grown on a DyScO_3_ substrate achieves a maximum *U*_rec_ of 2.172 *W*_0_(~5.43 × 10^6^ J m^−3^), which is significantly higher than that of regular paraelectric SrTiO_3_ films (3.2 × 10^6^ J m^−3^). If *E*_BD_ reaches 10*E*_0_, i.e., ~960 kV cm^−1^, the estimated *U*_rec_ becomes significantly higher. The performance peak value observed in the superlattice structure grown on a TbScO_3_ substrate is a high *U*_rec_ of 2.777 *W*_0_(~6.94 × 10^6^ J m^−3^).

For superlattices grown on substrates with even larger lattice constants, the saturation polarization (*P*_max_) decreases, leading to a reduction in *U*_rec_. However, the linear hysteresis loops induced by the polarization rotation mechanism have very low hysteresis loss, which gives rise to high energy storage efficiency. The theoretically predicted energy efficiency is remarkably high; for substrates with a lattice constant larger than that of TbScO_3_, the efficiency exceeds 80%. Considering the recoverable energy density and energy efficiency, the ST_8_/BT_8_ superlattices on DyScO_3_ and TbScO_3_ substrates emerge as promising candidates for energy storage applications.

## 4. Summary

In summary, using phase-field simulations, we systematically investigated the ferroelectric domain structures and polarization dynamics in (SrTiO_3_)_8_/(BaTiO_3_)_8_ superlattices under various substrate strains. The following are the most important findings:

(1) A substrate-electric field phase diagram was established, and we identified six typical simulated domain patterns. This study shows that stable and topologically protected skyrmions can be achieved via substrate engineering and external field control.
(2) Two distinct domain evolution modes under an electric field for different substrates were discovered. In Mode I, the domain structure evolution undergoes three distinct stages with an increasing electric field: labyrinth, skyrmion, and monodomain. In Mode II, the variation of polarization is primarily caused by the rotation of the polarization vectors.
(3) We revealed that the substrate mismatch strain effectively modulates the hysteresis loops, thereby endowing the (SrTiO_3_)_8_/(BaTiO_3_)_8_ superlattices with relatively high recoverable energy density *U*_rec_ and efficiency *η*. Our simulation results show that the (SrTiO_3_)_8_/(BaTiO_3_)_8_ superlattices on DyScO_3_ and TbScO_3_ substrates emerge as promising candidates for energy storage applications.

These results highlight the potential of strain engineering for designing next-generation nanoscale ferroelectric materials targeting both topological nanoelectronics and high-performance energy storage applications. Further experimental studies are needed to validate the theoretical predictions presented here and to facilitate the development of future nanoscale ferroelectric devices.

## Figures and Tables

**Table 1:** Lattice parameters of the substrate and the calculated misfit strain in the ST and BT layers.

Substrate	Lattice Parameter (Å)	Strain in BT	Strain in ST
SrTiO_3_	3.905	−2.38%	0.00%
DyScO_3_	3.945	−1.38%	1.02%
TbScO_3_	3.959	−1.03%	1.38%
GdScO_3_	3.968	−0.80%	1.61%
SmScO_3_	3.986	−0.35%	2.07%
BaTiO_3_	4.0001	0.00%	2.44%

**Table 2:** Landau energy coefficients, gradient energy coefficients, elastic energy coefficients and electrostrictive coefficients used in this work; T in Kelvin.

Parameter	BaTiO_3_	SrTiO_3_	Unit
*α*_1_	4.124(T−388) × 10^5^	2.6353[coth(42/*T*) − 0.90476] × 10^7^	C^−2^·N·m^2^
*α*_11_	−2.097 × 10^8^	1.696 × 10^9^	C^−4^·N·m^6^
*α*_12_	7.974 × 10^8^	1.373 × 10^9^	C^−4^·N·m^6^
*α*_111_	1.294 × 10^9^	0	C^−6^·N·m^10^
*α*_112_	−1.950 × 10^9^	0	C^−6^·N·m^10^
*α*_123_	−2.500 × 10^9^	0	C^−6^·N·m^10^
*α*_1111_	3.863 × 10^10^	0	C^−8^·N·m^14^
*α*_1112_	2.529 × 10^10^	0	C^−8^·N·m^14^
*α*_1122_	1.637 × 10^10^	0	C^−8^·N·m^14^
*α*_1123_	1.367 × 10^10^	0	C^−8^·N·m^14^
*G*_11_	3.46 × 10^−10^	3.46 × 10^−10^	C^−2^·N·m^4^
*G*_12_	0	0	C^−2^·N·m^4^
*G*_44_	1.73 × 10^−10^	1.73 × 10^−10^	C^−2^·N·m^4^
*C*_11_	1.78 × 10^11^	3.36 × 10^11^	N·m^−2^
*C*_12_	0.964 × 10^11^	1.07 × 10^11^	N·m^−2^
*C*_44_	1.22 × 10^11^	1.27 × 10^11^	N·m^−2^
*Q*_11_	0.10	0.066	C^−2^·m^4^
*Q*_12_	−0.034	−0.0135	C^−2^·m^4^
*Q*_44_	0.029	0.0096	C^−2^·m^4^

## Data Availability

The original contributions presented in this study are included in the article. Further inquiries can be directed to the corresponding authors.

## Abbreviations

The following abbreviations are used in this manuscript:

BT	Barium Titanate (BaTiO_3_)
ST	Strontium Titanate (SrTiO_3_)
PT	Lead Titanate (PbTiO_3_)
